# Role of mTOR Signaling in Female Reproduction

**DOI:** 10.3389/fendo.2019.00692

**Published:** 2019-10-09

**Authors:** Zaixin Guo, Qi Yu

**Affiliations:** Department of Obstetrics and Gynecology, Peking Union Medical College Hospital, Peking Union Medical College and Chinese Academy of Medical Sciences, Beijing, China

**Keywords:** mTOR signaling, follicular development, oocyte maturation, ovulation, steroidogenesis, gonadotoxicity, polycystic ovarian syndrome (PCOS), endometriosis

## Abstract

Mammalian target of rapamycin (mTOR) is a conserved serine/threonine kinase of the phosphatidylinositol kinase-related kinase family that regulates cell growth, metabolism, and autophagy. Extensive research has linked mTOR to several human diseases including cancer, neurodegenerative disorders, and aging. In this review, recent publications regarding the mechanisms underlying the role of mTOR in female reproduction under physiological and pathological conditions are summarized. Moreover, we assess whether strategies to improve or suppress mTOR expression could have therapeutic potential for reproductive diseases like premature ovarian failure, polycystic ovarian syndrome, and endometriosis.

## Introduction

Rapamycin is a macrocyclic lactone produced by the bacterium *Streptomyces hygroscopicus* that was previously used as an antifungal agent but abandoned soon after because of its immunosuppressive effect (1, 2). The protein target of rapamycin (TOR) was originally discovered in genetic mutation studies using *Saccharomyces cerevisiae* (3) and subsequently found to be the target of the rapamycin–FKBP12 complex in mammalian cells, which is now referred to as the mammalian target of rapamycin (mTOR) (4–6). mTOR is sensitive to diverse environmental inputs including nutrients and growth factors, and regulates various fundamental cell processes including cell growth, metabolism, differentiation, and autophagy (7). The dysregulation of mTOR has been observed in many diseases like cancer (8), diabetes (9), neurodegenerative disorders (10), and aging (10). Further, targeting mTOR is one of the most promising fields for the efficient treatment of these diseases.

Cellular metabolism comprises the foundation of all biological activities including female reproduction (11, 12). Recently, accumulating lines of evidence have shown that mTOR-regulated processes are important for folliculogenesis (13), oocyte meiotic maturation (14), ovarian somatic cell proliferation and steroidogenesis (15), puberty onset (16), ovarian aging (17), endometrium changes (18), and embryonic development (19). In this review, the role of mTOR signaling in female reproduction will be discussed, and the data describing alterations to this pathway under pathological conditions will be summarized.

## Brief Overview of mTOR Signaling

mTOR forms the catalytic subunit of two different multi-molecular complexes known as mTOR complex 1 (mTORC1) and mTOR complex 2 (mTORC2) (20). mTORC1, characterized by its sensitivity to rapamycin treatment, consists of mTOR, Raptor, mLST8/GβL, PRAS40, and DEPTOR. mTORC2, which is insensitive to acute rapamycin treatment but is inhibited by prolonged usage (21), is composed of mTOR, Rictor, mLST8/GβL, DEPTOR, mSin1, and protor1/2 (22).

mTORC1 plays a vital role in coordinating several cellular processes to help cells grow and divide. These processes include protein synthesis, lipid and nucleotide biogenesis, and autophagy (22). The critical effectors downstream of mTORC1 involving protein synthesis are p70S6 Kinase 1 (S6K1) and eIF4E Binding Protein 1 (4EBP1) (23), which deserve special emphasis for their important roles in female reproduction as described in the following sections. The cellular pathways upstream of mTORC1 include growth factors, stress, and amino acids (22). Briefly, numerous growth factor pathways inhibit tuberous sclerosis complex (TSC) (24, 25), which is composed of TSC1, TSC2, and TBC1D7 (26), relieve the inactivation of Ras homolog enriched in brain (Rheb) (27–30), and stimulate mTORC1 kinase activity. Certain incompatible stresses such as hypoxia, low ATP levels, or DNA damage, activate AMP-activated protein kinase (AMPK) pathway, which inhibits mTORC1 via the phosphorylation of Raptor (31) or TSC2 (32–34). For amino acids, there are two distinct pathways that stimulate mTORC1, and these are dependent or independent of the Rag GTPases (35–37).

The most important function of mTORC2 is the phosphorylation of Akt, which subsequently affects cell growth and proliferation (38). Moreover, mTORC2 activates several members of the AGC family, regulating cytoskeletal remodeling and cell migration (39–41). The cellular pathways upstream of mTORC2 are insulin/PI3K and mTORC1 (42, 43).

## mTOR Signaling in Folliculogenesis

During folliculogenesis, primordial follicles develop to primary, preantral, antral, and preovulatory stages, and are finally able to release an oocyte for fertilization (44–47). Primordial follicle activation, which is the beginning of follicular development after puberty, determines ovarian reserve and reproductive lifespan (44). mTOR signaling is involved in these changes. To investigate the role of mTOR signaling in this process, genetically modified mice were used in which one or several stages of different cells could be conditionally modified and specifically distinguished (Table 1). In this study, different conditional knockout (cKO) mouse models were named as follows: Gdf9–CRE-mediated cKO in primordial oocytes and all subsequent oocyte stages, referred to as “OogKO”; Zp3–CRE-mediated cKO in growing oocytes, referred to as “OozKO”; Foxl2–CRE-mediated cKO in primordial follicle granulosa cells, referred to as “pfGCKO”; AMHR2–CRE-mediated cKO, referred to as “Amhr2KO”; CYP19-CRE-mediated cKO, referred to as “Cyp19KO.” AMHR2 is mainly expressed in granulosa cells from preantral and small antral follicles, with little or no expression in the corpora luteum, large antral follicles, primordial follicles, and oocytes. Moreover, AMHR is also expressed in the fetal Müllerian duct mesenchyme and ovarian surface epithelium (48, 49). CYP-19 expressed in GCs of antral follicles and luteal cells (50).

**Table 1:** Ovarian-specific effects of altered mTOR signaling.

**Mutant mice**	**Tsc2-OogKO**	**Tsc1-OogKO**	**Tsc1-PfGCKO**	**Tsc1-Amhr2KO**	**Tsc1-Cyp19KO**	**Mtor-OogKO**	**Mtor-OozKO**	**Raptor-OogKO**	**Raptor-pfGCKO**
Reference	Adhikari et al. (51)	Adhikari et al. (52)	Zhang et al. (59)	Tanaka et al. (49)	Huang et al. (66)	Guo et al. (14)	Guo et al. (14)	Gorre et al. (57)	Zhang et al. (59)
mTOR signaling	Activated	Activated	Activated	Activated	Activated	Suppressed	Suppressed	Suppressed	Suppressed
Fertility	Infertility after 12~13 weeks of age	Infertility after 12~13 weeks of age	No data	Complete infertility	↑pups/litter	Complete infertility (naturally); ↓rate of fertilization in IVF	Nearly complete infertility (naturally); ↓rate of fertilization in IVF	Normal fertility	No data
Estrous cycles	No data	No data	No data	↑estrous cycle length, ↓diestrus cycle length	No data	No data	No data	No data	No data
Sex hormones	No data	↑FSH and LH at 3 and 4 months	No data	No change in E2 and P4 at diestrus and estrus	No data	↓E2 and P4 at 6 months	No change in E2 and P4 at 6 months	No data	No data
Follicle population and health	Follicle populations normal at PD13; all primordial follicles activated at PD23 & PD35; almost all follicles degenerated at 4 months	Follicle populations normal at PD5; all primordial follicles activated at PD23 & 7 weeks; almost all follicles degenerated at 2 and 3 months	Follicle populations normal at PD10; all primordial follicles activated at PD23 & PD35; almost all follicles degenerated at 4 months	Follicle populations normal at 6 weeks; ↓primordial follicles and ↑atretic follicles at 12 and 24 weeks; CL normal	↑growing follicles and antral follicles at 6 weeks; CL normal at 6 weeks; ↑CL at 3 and 6 months	↓large secondary and ↑primary follicles at PD21; ↓normal follicles at 3 months; no normal follicles at 6 months	Follicle populations normal at PD21 and 6 months	Follicle populations normal at PD35 & 16 weeks; CL normal at 16 weeks	Follicle populations normal at PD5; most follicles stay in primordial follicles at PD13 & PD35; no normal follicles at 4 months
Ovulation and oocyte health	No data	No data	No data	↑naturally released oocytes but 83% degenerated oocytes; same superovulated oocytes but 7-fold increase in degenerated oocytes	↑naturally released and superovulated oocytes	↓superovulated oocytes but 78.2% oocytes display incomplete cytokinesis or improper progression of meiosis to MII	Similar superovulated oocytes but 65.5% oocytes display incomplete cytokinesis or improper progression of meiosis to MII	No data	No data
Embryo development	No data	No data	No data	↑E2.5 embryos but 2/3 degenerated bodies; E3.5 embryos stay in ampullas	No data	↓progression to 2-cell and blastocyst stage in IVF	↓progression to 2-cell and blastocyst stage in IVF	No data	No data

mTOR signaling in oocytes is an important mechanism of primordial follicle activation, but is not necessary for the transition from primordial to primary follicle. As mentioned, TSC1 and TSC2 are negative regulators of mTORC1. A specific deletion of *Tsc1* (Tsc1-OogKO) or *Tsc2* (Tsc2-OogKO) genes in mouse oocytes of all stages results in the global awakening of oocytes at post-natal day (PD) 23 and depletion at 4 months of age. Enhanced mTOR signaling caused by mutation activates S6K1 signaling and promotes protein translation, leading to the overactivation of primordial follicles (51, 52). In particular, Tsc/mTORC1 signaling in primordial follicles is independent of PTEN (phosphatase and tensin homolog deleted on chromosome 10)/PI3K (phosphatidylinositol 3 kinase) signaling (52), despite being proposed to be a PTEN/PI3K downstream pathway in some cell types (53). Moreover, mTOR was suggested to be the functional pathway of some drugs that act on primordial follicles. Drugs such as TGF-βR1 and AMPK inhibitors (54, 55) activate primordial follicles by stimulating mTOR signaling in oocytes, and LKB1 restrains primordial follicle activation by suppressing of the mTOR pathway (56). Despite facilitating the activation of primordial follicles, mTOR signaling is not indispensable for the transition from primordial follicles to primary follicles. In mTOR-OogKO female mice, more primary and fewer large secondary follicles with normal primordial follicles were shown in the ovaries at PD23. Further, the long term lack of mTOR disrupts follicular development, as it was found that there are essentially no normal follicles and many abnormal follicles that strikingly resemble testicular seminiferous tubules at 6 months of age (14). Mice with Raptor depletion in all oocytes (Raptor-OogKO) were found to exhibit similar follicular development and female fertility as WT (wild-type) mice (57). Comparing mTOR-OogKO mouse models, only mTORC1 signaling in oocytes was found to be inhibited in Raptor-OogKO mice, and compensatory elevation of PI3K signaling was proposed to the reason for unaffected follicular development (57).

mTOR signaling in primordial follicle granulosa cells can either activate primordial follicles or impede their transition to primary follicles. Similar to Tsc1-OogKO and Tsc2-OogKO models, mice with a targeted deletion of *Tsc1* in primordial follicle granulosa cells (Tsc1-PfGCKO) present with the premature awakening of dormant oocytes at PD23 and almost all follicles are degenerated at 4 months of age (58, 59). Further experiments showed that the overactivation of mTORC1 signaling in pfGCs activates KIT in oocytes through KIT ligand (KITL), which triggers a PTEN/PI3K/AKT (thymoma viral proto-oncogene 1)/FOXO3 (Forkhead box O3) cascade and awakens of dormant oocytes. As KITL is the key point of follicle activation, drugs like MAPK3/1 inhibitors could be used to preserve the ovarian reserve by inhibiting mTORC1–KITL signaling in pfGCs (60). Raptor-pfGCKO female mice display suppressed follicle activation at PD13 and PD35, and oocytes eventually die at approximately 4 months of age (59), indicating that the KIT-PI3K cascade in oocytes is indispensable for primordial follicle survival.

Suppressed mTOR signaling in the oocytes of growing follicles has relatively minor effects on the follicular development but alters the oocyte transcriptome and proteome. When mTOR is specifically knocked out in growing follicles (mTOR-OozKO), follicular development and the ovulation rate are virtually the same as those in WT mice. However, the oocyte meiotic progression and developmental competence of released oocytes is impaired (14). To explain the impairment, the transcriptome of fully grown GV (germinal vesicle) oocytes and the proteome of ovulated oocytes was tested. Here, 85 transcripts and 237 proteins were found to be differentially expressed in Mtor-ZcKO oocytes. Particularly, down-regulated proteins were involved in the processes “mRNA metabolic process” and “actin filament bundle assembly” (14). At the completion of oocyte growth, transcription is actively silenced and protein translation slows substantially such that selective proteins are stored during oocyte growth (61). Thus, these defects, after ovulation, reflect the differences during folliculogenesis, although follicular growth appears to be the same between mTOR-OozKO and WT mice.

Activation of mTOR signaling in granulosa cells promotes follicular development as FSH-stimulated differentiation of GCs is necessary for follicular growth (62, 63). In GCs *in vitro*, activity of hypoxia-inducible factor-1α (HIF-1α) stimulated by FSH is mediated by mTOR signaling, and HIF-1α is necessary for FSH-enhanced induction of multiple follicular differentiation markers and facilitates follicular differentiation to a preovulatory phenotype (64, 65). There are two mutant mice with stimulated mTOR signaling at different stages of GCs, presenting with distinct effects on follicular growth. Tsc1- Cyp19KO mice have more growing follicles in the ovary, more released oocytes, and give birth to more pups than control animals (66), indicating the catalytic role of elevated mTOR signaling. However, Tsc1-Amhr2KO mice have fewer primordial follicles but more atretic follicles in the ovary, as well as a similar number of healthy but more degenerated ovulated oocytes. The number of good-quality embryos was found to be the same as that in WT animals after natural mating which was determined by puncturing ampullas as the oviducts of Tsc1-Amhr2KO mice are blocked (49). As AMH is only expressed in granulosa cells from preantral and small antral follicles, it was speculated that increased mTOR signaling promotes enhanced follicular growth but many of these are finally atretic without sufficient mTOR in large antral follicles. Moreover, unhealthy oocytes is probably from previous ovulation accumulated in the blocked oviducts as healthy oocytes and embryos were the same as those in WT mice (49). However, these contentions require more experimental evidence.

## mTOR Signaling During Oocyte Meiotic Maturation

Immature oocytes maintain meiotic arrest until stimulation, which could comprise LH in mammals or progesterone/insulin/IGF-1 in frogs, and then resume the meiotic cell cycle. The following events include nuclear envelope breakdown (NEBD), chromatin condensation, first meiotic spindle formation (metaphase I, MI), and the first polar body extrusion (metaphase II, MII) (67, 68). During these processes, the localization of mTOR changes with time. mTOR is observed in the cytoplasm at the GV stage, around the chromosomes at NEBD, and on the spindle during MI–MII stages (69). To distinguish mTORC1 and mTORC2, raptor and rictor are also localized separately. Raptor co-localizes with mTOR on the spindle but rictor is expressed on the spindle poles of MI oocytes (69), indicating that mTORC1 and mTORC2 have different contributions to meiotic division.

Oocyte meiotic maturation encompasses the activation of translation and increases in overall protein synthesis, which has a close relationship with mTORC1 signaling. mTORC1 takes part in protein synthesis relying on S6K1 and 4EBP1. S6K1 relays the signal comprising the decision of whether to prepare for mRNA translation (70). 4EBP1 contributes to overall cap-dependent translation. Specifically, formation of the cap-binding protein complex is inhibited when hypo-phosphorylated 4EBP1 binds eIF4E (70). Although both signals are downstream of mTORC1, they exhibit divergent expression with respect to species variation. Moreover, rapamycin also displays different effects. In *Rana dybowskii* oocytes, S6K1 activity increases after progesterone stimulation and rapamycin blocks progesterone-induced oocyte GVBD in a dose-dependent manner (71). In *Xenopus*, although S6K1 and 4EBP1 are stimulated by progesterone or insulin, oocyte meiotic maturation and the activation of overall protein synthesis are unaffected by rapamycin (72, 73). This phenomenon could be explained by the effect of *Xenopus* oocytes' cap-independent translation mechanism (74), but whether rapamycin has other effects on oocytes has not been explored. In mammalian oocytes, 4EBP1 becomes gradually phosphorylated during meiotic maturation, which was shown in bovine, porcine, and murine models (75–77). S6K1 is already highly phosphorylated at the GV stage and significantly decreases in murine oocytes (76, 78), which occurs to conserve energy for costly cell cycle processes (79). In mouse oocytes, mTOR-4EBP1-eIF4E inhibitors including rapamycin and eIF4E antibodies do not block meiotic progression, but lead to abnormalities in spindle morphology and chromosome alignment, in turn resulting in chromosomal aneuploidy (78). This indicates that the disruption of mTOR signaling only downregulates the translation of specific mRNAs involved in spindle assembly and chromosomal alignment but does not influence overall translations (78). Other mechanism could explain other translation-associated effects (80).

Moreover, the actin cytoskeleton mediates various vital functions during oocyte meiotic maturation (81, 82), which is related to mTORC2 signaling (39). mTORC2 functions upstream of Rho GTPases to regulate the actin cytoskeleton (39), which participates in various events during meiotic maturation (81). Upon disrupting both mTORC1 and mTORC2 with prolonged rapamycin treatment or an mTOR antibody in mouse oocytes, spindle migration and asymmetric division are inhibited (83), which was found to be actin-dependent (81, 82).

## mTOR Signaling in Ovarian Somatic Cells

The expression of mTOR and associated signaling components in granulosa cells (GCs), theca-interstitial cells (TICs), and luteal cells is well-documented. These cells respond to many factors to proliferate and produce steroid hormones. Of these factors, gonadotropins such as follicle stimulate hormone (FSH), luteinizing hormone (LH), and human chorionic gonadotropin (hCG) have the most important role.

mTOR signaling regulates GC proliferation in response to FSH stimulation and TIC proliferation stimulated by LH/hCG. As the mTOR pathway regulates meiotic processes, meiosis is also modulated by mTOR based on its effects on cell cycle regulatory protein synthesis (84). Under *in vitro* FSH stimulation, rat GCs increased and mTOR signaling is enhanced in a dose-dependent manner (85). Further, proliferation can be improved by increasing of *cyclin D2* mRNA expression, which regulates the progression of the cell cycle (86). Rapamycin was found to suppress these process significantly (85). Further studies proved that FSH uses a cAMP/PKA (protein kinase A)/ERK-dependent pathway to stimulate mTOR signaling (85). It is worth mentioning that immortalized rat GCs are arrested in the G1 cell cycle stage by applying rapamycin, which also reveals mTOR functions in the cell cycle (87, 88). The proliferation of rat TICs *in vitro* is stimulated by LH/hCG and is mTOR-dependent, which was shown based on the expression of proliferative markers like CDK4, cyclin D3, and PCNA. In contrast to GCs, the proliferation of TICs occurs via the cAMP/PI3K/AKT cascade, and not the PKA/ERK pathway (89). Similarly, insulin also stimulates TIC proliferation via activation of the mTOR pathway (90).

mTOR signaling also participates in steroidogenesis. In TICs, the inhibition of mTORC1 by rapamycin (20 nM) was found to reduce the expression *Cyp11a1, Hsd3b1*, and *Cyp17a1* mRNA and the production of androstenedione in response to hCG. CREB (cAMP response element-binding protein) acts as downstream of S6K1 and mediates changes in gene expression in response to gonadotropin (15, 91). In human granulosa lutein cells, pre-treatment with rapamycin (20 nM) inhibits the hCG-induced upregulation of *Cyp11a1, Hsd3b1*, and *Star* mRNA and the production of progesterone *in vitro* (92). However, in bovine luteal cells, despite being activated by LH, mTOR signaling does not contribute to corpus luteum (CL)-derived progesterone synthesis (93).

Although mTOR signaling is speculated to be involved in luteal cell autophagy, further experiments disproved this. CL regression occurs if fertilization does not occur, and an important mechanism underlying the function of the CL is luteal cell autophagy (94). Although mTOR signaling is presumed to play a part in CL regression and stimulating the response to PGF2α (95), an important mediator of CL regression through increased ERK1/2 activity (96), another study suggested that luteal cell autophagy is induced by enhanced ERK1/2 activity and is independent of mTOR activity (97).

## mTOR Signaling in Puberty Onset, Fertility, and Ovarian Aging

mTOR signaling is highly expressed in the hypothalamus (98), and affects puberty onset when it is suppressed. After blocking central mTOR signaling via the intracerebroventricular administration of rapamycin in rats, puberty onset was found to be inhibited, which presented as decreased LH and estradiol levels, delayed vaginal opening, and atrophied ovaries and uterus (16). As mTOR signaling stimulates protein synthesis and inhibits autophagy in the presence of nutrients (98), hypothalamic mTOR signaling was proposed to play a part in the metabolic regulation of female puberty. The mechanism underlying the role of mTOR in puberty onset was implied by the fact that rapamycin suppresses arcuate nucleus (ARC) Kiss1 levels (16), the upstream regulator of GnRH release, which controls puberty onset (99). Interestingly, mTOR signaling seems to regulate Kiss1 neurons indirectly, as pS6 (the downstream of mTOR and S6K1) is not expressed in Kiss1 neurons (100). However, AMP-activated protein kinase (AMPK), another energy sensor activated by conditions of energy insufficiency (101), appears to be expressed in Kiss1 neurons, and has a putative role in the interplay between mTOR signaling and puberty onset (102). Although the significant effects of rapamycin on puberty onset were determined in animal experiments, patients clinically treated with rapamycin before menarche were found to have a similar menarche time as patients administered rapamycin post-menarche (103). The difference could be explained by the fact that rapamycin administered orally has little effects on mTOR signaling in the brain (104), although rapamycin is believed to cross the blood–brain barrier (105), and reduce depression and anxiety (106, 107).

Rapamycin disrupts menstruation and ovulation, leading to infertility both in animals and humans. As mentioned, mTOR signaling is indispensable for follicular development and the effects of rapamycin on fertility are easily demonstrated. Rapamycin impedes ovulation and affects menstruation *in vivo*. Mice exhibit irregular estrous cycles when administered rapamycin (5 mg/kg every other day or 2 mg/kg every day, i.p.) (17, 108). Serum analysis also revealed that progesterone (P4) is decreased dramatically (108). Further, ovarian morphological assessments demonstrated an increased number of primordial follicles and a decreased number of all growing follicles and the CL. During a superovulation regiment, the injection of rapamycin (5 or 50 mg/kg body weight for 4 days, i.p.) led to a dose-dependent decrease in the numbers of eggs released (88). Moreover, rapamycin-treated mice had no pregnancies during the mating trial (17). In the clinic, patients administered rapamycin present with similar phenomena. Rapamycin increases the risk of oligomenorrhea and/or ovarian cysts in patients with tuberous sclerosis complex (103), transplantation (109), and autosomal dominant polycystic kidney disease (110). In addition, the percentage of patients who experienced at least one menstrual irregularity was determined to be as high as 38.4% in the tuberous sclerosis complex study with 112 patients (103). Interestingly, all disturbances in menstruation returned to normal 2 months after stopping treatment (108), indicating that the function of rapamycin does not persist.

The inhibition of mTOR signaling can also prolong ovarian lifespan. Compared to that in control rats, rapamycin-treated rats have a 2-fold increase in the number of primordial follicles after 10 weeks of treatment with rapamycin (5 mg/kg every other day, i.p.) (17, 111). These results indicate that rapamycin can protect the ovarian reserve and extend ovarian lifespan. An interesting experiment also showed that a transient 2-weeks regimen of rapamycin facilitates the sufficient extension of ovarian lifespan in mice regardless of the age at treatment initiation (108). Although improvements in oocyte quality and the ovarian microenvironment were demonstrated experimentally, this requires more exploration to identify the relationship between treatment time and the extension of ovarian lifespan.

## mTOR Signaling in the Endometrium

The endometrium undergoes cyclical and rhythmic changes under the influence of complex autocrine, paracrine and endocrine signaling (112). Further, this is a process involving cell proliferation, differentiation, apoptosis, autophagy, and decidualization, in which mTOR signaling plays a role. Estradiol-17β (E2) and P4 are the most important factors orchestrating cell division and differentiation of the endometrium. E2 regulates protein synthesis and DNA synthesis in uterine epithelial cells through the PKC (phospho-kinase C)/ERK/mTOR pathway, which finally manipulates cell proliferation. However, P4 inhibits only the E2-induced DNA synthetic response without affecting mTOR signaling, indicating another pathway responsible for P4 inhibition (18). The suppression of mTOR signaling also promotes human endometrial stromal cell (ESC) apoptosis via autophagy induction, which is mediated by S6K1 and can be determined based on LC3-II expression (113, 114). Decidualization, the transformation of endometrial stromal cells into specialized secretory decidual cells (115), is affected by nm23 via the PI3K-Akt-mTOR signaling pathways in mouse ESCs and human ESCs (116). Based on the vital roles of mTOR in modulating the endometrium, its influence on endometrial receptivity, which renders the endometrium suitable for embryo development (117, 118), is easily understood. Experiments have shown that activation of the ERK1/2-mTOR pathway is one mechanism through which fludrocortisone affects uterine receptivity in mice (119).

In addition, the mTOR pathway also participates in implantation. Levels of mTOR in the pregnant mice are higher than those in non-pregnant mice. Moreover, the levels are increased from PD3, reach a maximum on PD5, and then decline thereafter (120). Stimulation of the PI3K/PKB/mTOR/NO signaling pathway by dietary arginine supplementation can also enhance embryo implantation (121). mTOR is also indispensable for placentation in the porcine uterine tissue based on its important function during translation (122, 123).

## mTOR Signaling During Post-Fertilization Events

mTOR signaling is essential for embryonic development both *in vivo* and *in vitro*. In *Drosophila melanogaster*, cell size and embryonic lethality are reduced when TOR or S6 kinase is silenced (124, 125). In mice, disruption of mTOR completely results in the death of embryos shortly after implantation, which was attributed to impaired cell proliferation in both embryonic and extraembryonic compartments. This was proven by *in vitro* experiments in which both the inner cell mass (ICM) and trophoblasts fail to proliferate in mTOR-deleted mouse blastocysts (19). When mTOR is disrupted partly, mutant embryos die at midgestation coitum, which is accompanied by failed telencephalon cell growth and rotation around the embryonic body axis (126, 127). Different from that with mTOR, mice deficient in S6K1 are viable and smaller in body size, which could be explained by the fact that S6K1 only represents one part of mTOR signaling (128). Drugs that inhibit mTOR signaling impair blastocysts growth, but the effects are reversible. When treated with rapamycin (200 nM), growth of the ICM is not inhibited, but trophoblast outgrowth of blastocysts is impeded (19). Interestingly, the proliferation of embryonic stem cells is also refractory to rapamycin. An insufficient number of rapamycin receptors or the existence of mTORC2 could be the reason for this (19). Another exciting discovery is that the inhibition of both mTORC1 and mTORC2 complexes using INK128 or RapaLink-1 results in a diapause state of blastocysts. Diapause is a reversible pausing state triggered by unfavorable conditions (129, 130). When the inhibition is relieved, blastocysts can give rise to live, fertile mice (131).

mTOR signaling also takes part in mammalian embryonic differentiation, during which embryonic stem cells and pluripotent stem cells grow into the endoderm, ectoderm and mesoderm (132). Interestingly, mTOR signaling presents different functions in mouse embryonic stem cells and human pluripotent stem cells. In mice, the activation mTOR induces mouse embryonic stem cell differentiation. Increasing mTOR activity via withdrawal of the cytokine leukemia inhibitory factor from mouse embryonic stem cell culture can induce mouse embryonic stem cell differentiation (133, 134). Further, a decrease in mTORC2 activity, resulting from the knockdown of calcineurin, impairs mesoderm differentiation (135). In contrast to that in mouse embryonic stem cells, mTOR inhibition promotes human pluripotent stem cell differentiation. The knockdown of Raptor in human pluripotent stem cells induces mesendoderm differentiation (136), and the same effect occurs when treating human pluripotent stem cells with rapamycin (137). The mechanism underlying this difference between mice and humans is not understood, and additional studies are needed to explore this, as the cell populations are similar (138).

## Roles of mTOR Signaling in Female Reproductive Pathology

As mTOR signaling affects many processes associated with reproduction, manipulating this pathway to preserve fertility has been explored in preclinical studies. The pharmacologic downregulation of mTOR protects the ovarian reserve in the presence of ovarian toxic drugs in animal models (139), as mTOR is related to primordial follicle activation. In fact, anticancer therapies such as genotoxic or antimitotic agents hardly impair most oocytes in primordial follicles. However, when the ovary is repeatedly exposed to chemotherapy, primordial follicles are activated and grow to replace damaged growing follicles (140, 141). Thus, regimens to counteract follicle activation could prevent premature ovarian failure induced by toxicity. It was also confirmed that mTOR inhibitors can promote follicular quiescence and prevent premature ovarian failure when used in cyclophosphamide-induced and cisplatin-induced ovarian dysfunctional animal models (139, 142, 143).

Activating mTOR signaling is a vital mechanism used in primordial follicle growth activation (PFGA) (144), which has great potential to assist reproduction. PFGA is an explored technology that is expected to provide benefits for patients with diminished ovarian reserve or malignant tumors. One problem with hormonal stimulation strategies used clinically is that they cannot fully utilize most follicles remaining in the ovary such as primordial follicles but largely rely on the population of growing follicles. Thus, a critical step to fully exploit the ovarian follicle is to overcome the mechanism resulting in the growth-arrest of primordial follicles (144). mTOR activators (phosphatidic acid, propranolol) can induce the awakening of primordial follicles in the human ovarian cortex such that more mature follicles can be obtained in combination with other primordial follicle activators (145, 146).

Polycystic ovarian syndrome (PCOS) is a multifactorial endocrinopathy that affects reproduction and metabolism (147, 148). Many conclusions involving the relationship between PCOS and mTOR signaling are paradoxical and complex. For example, in a DHEA-induced PCOS mouse model, the expression of mTOR and p-mTOR (serine-2448) in the ovary was found to be higher than that in normal mice, but S6K1 is decreased in the DHEA-treated PCOS mouse ovary. With respect to this, the authors speculated that insufficient S6K1 activation causes an arrest in follicular development but they could not explain the discrepancy in expression between mTOR and downstream signaling (149). In another study, mTOR protein levels in luteal GCs of PCOS patients were the same as those in healthy women. However, less mTOR protein expression is observed in luteal GCs with PCOS compared to that in healthy patients upon stimulation with insulin (150). Therefore, further experiments are needed to delineate the accurate expression of mTOR in PCOS. Another characteristic linking PCOS and mTOR is metabolic disorder during PCOS. Rapamycin was found to enhance insulin sensitivity and improve serum lipid profiles after long term treatment (151). Thus, rapamycin is expected to be used to eliminate metabolic syndrome with PCOS despite potential adverse effects on fertility and short-term metabolism.

Endometriosis, a gynecological disease caused by the dislocation of endometrial cells, is characterized by inflammation and to progesterone resistance (152). Inadequate responses to progesterone in eutopic and ectopic endometrial cells and tissue contribute to increased cell proliferation (153), in which mTOR is involved. The ectopic endometrium of patients with endometriosis exhibits increased phosphorylation of mTOR compared to that in the eutopic endometrium (154–156). Moreover, the inhibition of mTOR can suppress endometriotic foci in a rat/mouse model of endometriosis (157, 158) and promote human endometriotic cell apoptosis via autophagy induction (113, 159). In addition to the ectopic endometrium, aberrant decidualization in the eutopic endometrium of women with endometriosis impairs implantation partially through the activation of PI3K/AKT (160, 161). As mTORC2 is downstream of AKT, it can be postulated that mTOR is activated in the eutopic endometrium, but this requires experiments for confirmation.

## Conclusions

**Figure 1:**
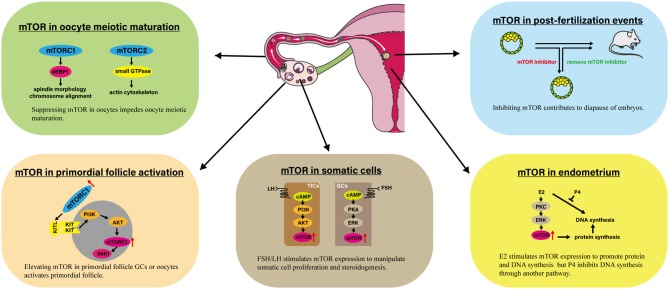
Involvement of mTOR in several processes linked to female reproduction. The impressive events are presented involving mTOR in primordial follicle activation, oocyte meiotic maturation, somatic cells, endometrium, and post-fertilization events. AKT, Protein kinase B; E2, Estradiol-17β; 4EBP1, eIF4E Binding Protein 1; ERK, Extracellular signal regulated kinase; KITL, KIT ligand; mTOR, Mammalian target of rapamycin; mTORC1, mTOR complex 1; P4, Progesterone; PI3K, Phosphoinositide 3-kinase; PKA, Protein kinase A; PKC, Protein kinase C; S6K1, p70S6 Kinase 1.

mTOR signaling plays a vital role in regulating female reproduction, which has been demonstrated based on data from genetic, pharmacological, and clinical studies (Table 2, Figure 1). mTOR signaling participates in various process that occur in the ovary, including ovarian reserve, follicle development, oocyte meiotic maturation, ovarian aging, and proliferation and steroidogenesis of ovarian somatic cells, among others. There are two impressive functions of mTOR signaling in the ovary. First, elevated mTOR expression in both oocytes and primordial follicle GCs activates the primordial follicle, which has great potential for applications of ovarian reserve protection and PFGA. Second, suppressing mTOR hinders oocyte meiotic maturation, which could limit the use of mTOR-suppressing drugs for fertility-related diseases. In addition to that in the ovary, mTOR appears to be crucial for hypothalamus functions, endometrium changes, and embryo development. Finally, preclinical evidence suggests the possibility of applying mTOR modulators to ameliorate fertility issues associated with POF, PCOS, and endometriosis.

**Table 2:** Role of mTOR signaling in female reproductive cells and organs.

**Cell type**	**Proposed function**	**Species**	**Proposed mediator**	**References**
Oocyte	Folliculogenesis	Mouse	No data.	(14, 49, 51, 52, 57)
	Maturation	Mouse, porcine, bovine	eIF4E, Rho GTPases	(39, 75–78, 83)
	Ovarian aging	Mouse	No data.	(17, 111)
Granulosa cells	Folliculogenesis	Rat, mouse	HIF-1α	(49, 64–66)
	FSH induced-proliferation	Rat	Cyclin D	(85, 86)
Luteal granulosa cells	Steroidogenesis	Human; not applicable for bovine	Cyp11a1, Hsd3b1, Star	(92, 93)
	Autophagy (proven to unrelated)	Rats	-	(95, 97)
Primordial follicle granulosa cells	Folliculogenesis	Mouse	KITL	(59)
Theca-interstitial cells	LH/hCG induced proliferation	Rat	CDK4, Cyclin D, PCNA	(89)
	Steroidogenesis	Rat	CREB, Cyp11a1, Hsd3b1, Cyp17a1	(15)
Hypothalamus	Puberty onset	Mouse	Kiss 1	(16)
Embryo	Embryo development	Mouse	No data	(19, 128–131)
	Embryo differentiation	Mouse, human	No data	(133–138)
Endometrium	Proliferation; apoptosis; autophagy	Mouse, human	No data	(18, 112–116)
	Implantation	Mouse	NO	(120, 121)
	Placentation	Porcine	No data	(122, 123)

Although rapamycin and catalytic mTOR inhibitors are already successfully used to prevent the rejection of transplants or treat some types of cancer, many studies are needed to translate experimental results to clinical use for female reproductive diseases. On one hand, mTOR seems to be much more than a simple positive or negative trigger of female reproduction. Under physiological conditions, mTOR is regulated by different factors in a joint effort to determine the outcome of several processes including folliculogenesis, ovulation, endometrium changes, or embryonic development. On the other hand, the complete inhibition of mTOR would cause severe dose-limiting toxicities based on the indispensable role of mTOR in most human tissues. Thus, it has yet to be determined if it is worthwhile to cure non-life threatening diseases with an mTOR inhibitor (162, 163). Future work should focus on the development of tissue-specific therapeutics to avoid drawbacks associated with effects on unrelated tissues. Overall, mTOR clearly plays an important role in female reproduction. However, much work is needed to fully understand the mechanisms underlying the role of mTOR in female reproduction and to completely unlock the therapeutic potential of this signaling pathway.

## Author Contributions

ZG collected the information, designed the figures, wrote the manuscript, and submitted the manuscript. QY contributed to manuscript conception, design, and critical discussion.

### Conflict of Interest

The authors declare that the research was conducted in the absence of any commercial or financial relationships that could be construed as a potential conflict of interest.
